# Efficient Online Object Tracking Scheme for Challenging Scenarios

**DOI:** 10.3390/s21248481

**Published:** 2021-12-20

**Authors:** Khizer Mehmood, Ahmad Ali, Abdul Jalil, Baber Khan, Khalid Mehmood Cheema, Maria Murad, Ahmad H. Milyani

**Affiliations:** 1 Department of Electrical Engineering, International Islamic University, Islamabad 44000, Pakistan; khizer.mehmood@iiu.edu.pk (K.M.); abdul.jalil@iiu.edu.pk (A.J.); baber.khan@iiu.edu.pk (B.K.); maria.murad@iiu.edu.pk (M.M.); 2 Department of Software Engineering, Bahria University, Islamabad 44000, Pakistan; ahmad.buic@bahria.edu.pk; 3 School of Electrical Engineering, Southeast University, Nanjing 210096, China; 4 School of Electrical and Computer Engineering, King Abdulaziz University, Jeddah 21589, Saudi Arabia; ahmilyani@kau.edu.sa

**Keywords:** object tracking, image processing, fractional-gain Kalman filter, APCE

## Abstract

Visual object tracking (VOT) is a vital part of various domains of computer vision applications such as surveillance, unmanned aerial vehicles (UAV), and medical diagnostics. In recent years, substantial improvement has been made to solve various challenges of VOT techniques such as change of scale, occlusions, motion blur, and illumination variations. This paper proposes a tracking algorithm in a spatiotemporal context (STC) framework. To overcome the limitations of STC based on scale variation, a max-pooling-based scale scheme is incorporated by maximizing over posterior probability. To avert target model from drift, an efficient mechanism is proposed for occlusion handling. Occlusion is detected from average peak to correlation energy (APCE)-based mechanism of response map between consecutive frames. On successful occlusion detection, a fractional-gain Kalman filter is incorporated for handling the occlusion. An additional extension to the model includes APCE criteria to adapt the target model in motion blur and other factors. Extensive evaluation indicates that the proposed algorithm achieves significant results against various tracking methods.

## 1. Introduction

**Figure 1:**
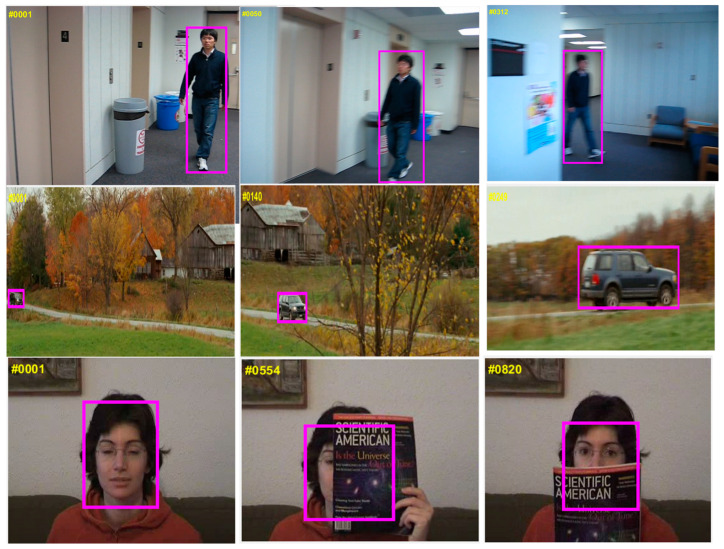
Challenging scenarios in visual object tracking (VOT). The first row shows motion blur in an image sequence. The second row shows the scale variation of the target. The third row shows heavy occlusion of the target. Pictures in the figure are part of OTB-100 dataset [26].

Visual object tracking (VOT) is an essential task in a variety of computer vision applications such as video surveillance [1,2,3], automobile [4], human–computer interaction [5], cinematography [6], sensor network [7], motion analysis [8], robotics [9,10,11], anti-aircraft system [12], autonomous vehicles [13], and traffic monitoring [14]. As presented in Figure 1, VOT remains a challenging issue due to motion blur, occlusion, fast motion, among others [15,16,17,18,19].

Tracking methods can be categorized as generative and discriminative. In generative tracking methods, the computation cost is high, and they are adaptable with environmental factors due to which these tracking methods might fail in background clutter situations [20,21,22]. Discriminative tracking methods perform better in clutter background situations since they treat these as a binary classification problem. However, they are slow, making them unsuitable for real-time applications [23,24,25].

### 1.1. Related Work

The STC tracker [27] has been widely used in recent years due to its computational efficiency. STC integrates spatial context information around the target of interest and considers prior information of previous frames for computing the extreme-of-confidence map by using Fourier transform. Die et al. [28] combined a correlation filter (CF) and STC. They extracted HOG (histogram of oriented gradients), (CN) color naming, and gray features for learning-correlation filters. Then, the response of CF and STC is fused. Yang et al. [29] proposed an improved tracking method by incorporating peak to sidelobe ratio (PSR)-based occlusion detection mechanism and model update scheme in the STC framework. Zhang et al. [30] proposed a tracking method by incorporating HOG, CN features, and an average difference of frames-based adaptive learning rate mechanism in the spatiotemporal context framework. Zhang et al. [31] suggested a tracking method by incorporating a selection update mechanism in the spatiotemporal context framework. Song et al. [32] anticipated an improved STC-based tracking method by combining a scale filter and loss function criteria for better performance in UAV applications.

During the past decade, significant progress has been made to develop accurate scale estimation in VOT [33,34,35,36,37,38]. Danelljan et al. [39] proposed a tracking-by-detection framework by learning filters for translation and scale estimation based on pyramid representation. Li et al. [40] incorporated an adaptive scale scheme in a kernelized correlation filter (KCF) tracker using HOG and CN features. Bibi et al. [41] modify the KCF tracker by maximizing posterior distribution over the scales grid and updating the filter by fixed point optimization. Lu et al. [42] combined KCF and Fourier–Mellin transform to deal with rotation and scale variation of the target. Yin et al. [43] modified the scale adaptive with multiple features (SAMF) tracker by using APCE-based rate of change between consecutive frames to control scale size. Ma et al. [44] incorporated APCE in discriminative correlation filters to address fixed template size.

A Kalman filter is used in various tracking algorithms for occlusion handling [45,46,47,48,49]. Kaur et al. [50] suggested a real-time tracking approach using a fractional-gain Kalman filter for nonlinear systems. Soleh et al. [51] proposed the Hungarian Kalman filter (HKF) for multiple target tracking. Farahi et al. [52] proposed a probabilistic Kalman filter (PKF) by incorporating an extra stage for estimating target position by applying the Viterbi algorithm to a probabilistic graph. Gunjal et al. [53] proposed a Kalman filter-based tracking algorithm for moving targets under surveillance applications. Ali et al. [54] address issues in VOT such as fast maneuvering of the target, occlusions, and deformation by combining Kalman filter, CF, and adaptive mean shift in the heuristic framework. Kaur et al. [55] proposed a modified fractional-gain-based Kalman filter for vehicle tracking by incorporating a fractional feedback loop and cost function minimization. Zhou et al. [56] address issues in VOT such as occlusions, motion blur, and clutter background by incorporating a Kalman filter in a compressive tracking framework.

By summarizing the current methods, it can be perceived that significant work has been done to develop a robust tracking algorithm by incorporating scale update schemes, model update mechanisms, occlusion detection, and handling techniques in different tracking frameworks. The STC algorithm proposed in [27] uses FFT for detection and context information for a model update. However, it cannot effectively deal with occlusions, scale variations, and motion blur.

### 1.2. Our Contributions

To address the limitations of the STC, this paper proposes a robust tracking algorithm suitable for various image processing applications, such as surveillance and autonomous vehicles. The contributions can be listed concisely as follows.

1. We introduce novel criteria for detecting occlusion by utilizing APCE, model update rules, and previous history of the modified response map to prevent the tracking model from wrong updates.
2. We introduce an effective occlusion handling mechanism by incorporating a modified feedback-based fractional-gain Kalman filter in the spatiotemporal context framework to track an object’s motion.
3. We incorporate a max-pooling-based scale scheme by maximizing over posterior probability in the STC framework’s detection stage. We applied a combination of STC and max-pooling to attain higher accuracy.
4. We introduce an APCE-based adaptive learning rate mechanism that utilizes information of current frame and previous history to reduce error accumulation and correctly updates from the wrong appearance of the target.
5. Extensive performance analysis of the proposed tracker is carried out on standard benchmark videos in comparison with STC [27], KCF_MTSA [41], MACF [57], MOSSE_CA_ [58], and Modified KCF [59].

### 1.3. Organization

The organization of this paper follows: brief explanations of STC and fractional calculus are provided in Section 2. In Section 3, the tracking modules of the proposed tracker are explained. Section 4 includes performance analysis. Discussion is given in Section 5, while Section 6 concludes the paper.

## 2. Review of STC and Fractional Calculus

### 2.1. STC Tracking

**Figure 2:**
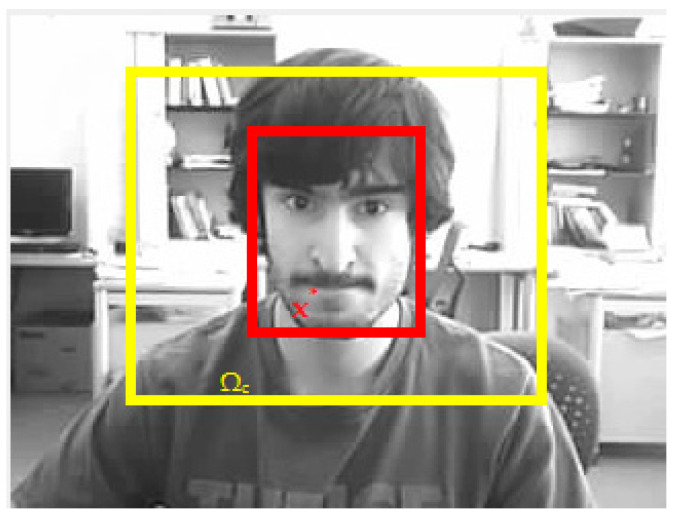
The spatial relation between object and its context. Picture in the figure is part of OTB-100 dataset [26].

The STC tracking algorithm formulates the relation between the target of interest and its context in the Bayesian framework. The feature set $X^{c}$ ={l(r)=(I(r),r)|r∈ Ω_c_(x*)} and spatial relation between target context is presented in Figure 2.

The confidence map is given as follows:

(1) $$\begin{array}{c} l\left(x\right)=\;P\left(x|k\right)=\underset{n\left(r\right)\;\in {\;X}^{c}}{\sum }P\left(x,l\left(r\right)|k\right)\\ =\sum_{l\left(r\right)\;\in {\;X}^{c}}^{}P\left(x,l\left(r\right)|k\right)P\left(l\left(r\right)|k\right) \end{array}$$

P(l(r)|k) is the prior context model and P(x,l(r)|k) is the spatial context model. The confidence map function l(x) is given in (2):

(2) $$l\left(x\right)=P\left(x|k\right)={\;v\;e}^{\left(-\left|\frac{x\;-{\;x}^{*}}{\emptyset }\right|\;Þ\right)}$$

where v is the normalization constant, Þ is a parameter for shape, and ∅ is a parameter for scale. The spatial context uses the intensity of the image and weighted Gaussian function given in (3) and (4):

(3) $$P\left(l\left(r\right)|k\right)=\;I\left(r\right)\omega_{\gamma }\left(r\;-{\;x}^{*}\right)$$

(4) $${\;\omega }_{\gamma }=\;\theta {\;e}^{\left(-\frac{{\left|x-x^{*}\right|}^{2}}{\sigma^{2}}\right)}$$

Equation (5) describes the spatial context model:

(5) $$P\left(x,l\left(r\right)|k\right)=h^{\mathrm{sc}}\left(x\;-\;r\right)$$

Explaining for the spatial context:

(6) $$={\;h}^{\mathrm{sc}}\left(x\;-\;r\right)I\left(r\right)\omega_{\gamma }\left(r\;-{\;x}^{*}\right)$$

(7) $$={\;h}^{\mathrm{sc}}\left(x\right)⊗\left(I\left(x\right)\omega_{\gamma }\left(x\;-{\;x}^{*}\right)\right)$$

Fast Fourier transform (FFT) can be calculated as follows:

(8) $$ℱ\left(l\left(x\right)\right)=ℱ\left(h^{\mathrm{sc}}\left(x\right)\right)⊙F\left(I\left(x\right)\omega_{\gamma }\left(x\;-{\;x}^{*}\right)\right)$$

The solution of (8) follows:

(9) $$h^{\mathrm{sc}}\left(x\right)={ℱ}^{-1}\left(\frac{F\left(v.e^{-\left|\frac{x\;-{\;x}^{*}}{\emptyset }\right|\;Þ}\right)}{F\left(\left(I\left(x\right)\omega_{\gamma }\left(x\;-{\;x}^{*}\right)\right)\right)}\right)$$

As presented in (10), $x_{t+1}^{*}$ can be obtained by computing the extreme-of-confidence map:

(10) $$x_{t+1}^{*}={\;\arg}_{x\in {Ω}_{c}\left(x_{t}^{*}\right)}{\max\;l}_{t+1}\left(x\right)$$

The confidence map can be considered from (11):

(11) $$l_{t+1}\left(x\right)={ℱ}^{-1}\left(F\left(H_{t+1}^{\mathrm{stc}}\left(x\right)\right)⊙ℱ\left(I_{t+1}\left(x\right)\omega_{\gamma }\left(x-{\;x}_{t}^{*}\right)\right)\right)$$

Spatiotemporal context is updated on learning rate ρ, as given in (12):

(12) $$H_{t+1}^{\mathrm{stc}}=\left(1-\;\rho \right)H_{t}^{\mathrm{stc}}+{\rho h}_{t}^{\mathrm{sc}}$$

### 2.2. Fractional Calculus

In this work, the Grünwald–Letnikov definition [60] is used for calculating fractional difference defined in (13):

(13) $$\;\;\;\;\;\Delta^{\gamma }{\;x}_{k}=\frac{1}{h^{n}}\;\overset{k}{\underset{q=0}{\sum }}{\left(-1\right)}^{q}\;\left(\begin{array}{c} n\\ q \end{array}\right){\;x}_{k+1-q}$$

where n is fractional order, h is the sampling interval, k is the number of samples of given signal x, and $\left(\begin{array}{c} n\\ q \end{array}\right)$ is obtained using (14):

(14) $$\left(\begin{array}{c} n\\ q \end{array}\right)=\{\begin{array}{cc} 1 & \mathrm{for}\;q=0\\ \frac{n\left(n-1\right)\ldots \left(n-q+1\right)}{q} & \mathrm{for}\;q>0 \end{array}$$

## 3. Proposed Solution

**Figure 3:**
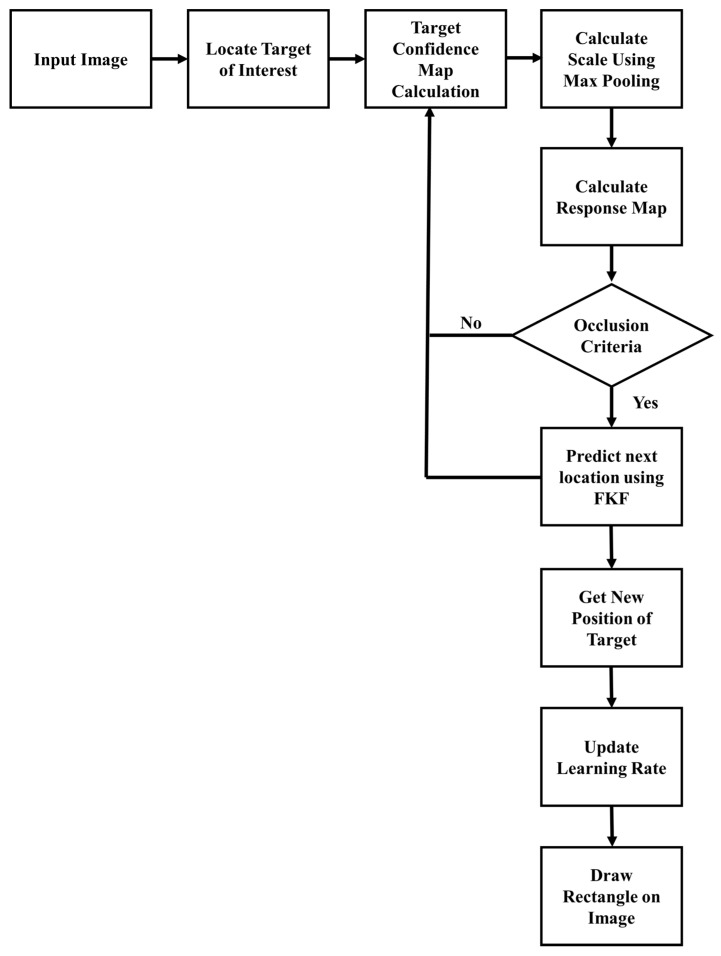
Flowchart of proposed tracking method.

In this section, tracking modules are elaborated. First, the max-pooling-based scale mechanism is presented. Second, the APCE-based occlusion detection mechanism is discussed. Third, the fractional-gain Kalman filter-based mechanism for occlusion handling is examined. Fourth, an APCE-based modified learning rate mechanism is explained. The flowchart of the proposed tracker is displayed in Figure 3.

As presented in Figure 3, for each sequence, the ground truth of the target is manually initialized in the first frame. Afterward, the confidence map of the target is calculated. Then, by maximizing the posterior probability, the scale of the target is estimated. Then APCE of the response map is calculated along with the difference of APCE between consecutive frames. Based on occlusion criteria, the fractional-gain Kalman filter activates and predicts the location of the target. Afterward, the learning rate of the tracking model is updated by utilizing the current target position and previous history of APCE values.

### 3.1. Scale Integration Scheme

One limitation of STC is the inability to rapidly change the scale. During the detection phase of STC, we applied max-pooling over multiple scales by maximizing the posterior probability, as given in (15):

(15) $$\underset{i}{\max}{\;P\;(r}_{i}|y)=P\;\left({y|r}_{i}\right)\;P\;\left(r_{i}\right)\;$$

where $r_{i}$ represents ith scale and $P\;\left({y|r}_{i}\right)$ is the maximum detection likelihood response at ith scale. The prior term $P\;\left(r_{i}\right)$ is the Gaussian distribution whose standard deviation is set through experimentation. It allows for a smooth transition between frames, given that the target scale does not vary much between frames.

### 3.2. Occlusion Detection Mechanism

The performance of any tracking algorithm is affected by various factors, of which the most common is occlusion. It is essential to create a mechanism for the detection of occlusion. In the present work, an occlusion feedback mechanism is presented, which detects occlusion and updates the target model by evaluating the tracking status of each frame.

Average peak to correlation energy (APCE) [61] determines tracker effectiveness. The value of APCE changes according to the target occlusion state. Small values of APCE specify tracking failure or target occlusion. It is given in (16):

(16) $${\mathrm{APCE}}_{t}=\frac{{\left|g_{\max\;}-{\;g}_{\min}\right|}^{2}}{\mathrm{mean}\left(\sum_{w,h}{\left(g_{w,h}-{\;g}_{\min}\right)}^{2}\right)}$$

where $g_{\max\;}$and${\;g}_{\min}$ are maximum and minimum response values, respectively, and $g_{w,h}$ gives indices of the response map. The occlusion detection criteria are built as given in (17) and (18):

(17) $$\delta ={\mathrm{APCE}}_{t}-{\mathrm{APCE}}_{t-1}$$

(18) $${\mathrm{APCE}}_{t}<\epsilon_{\mathrm{th}}$$

where ${\mathrm{APCE}}_{t}$ and ${\mathrm{APCE}}_{t-1}$ are the APCE values at t and (t − 1) frames, respectively, δ is the difference of the APCE between two sequential frames, and $\epsilon_{\mathrm{th}}$ is the threshold value acquired by performing multiple experiments. Rules of occlusion and model update follow:

1. When δ≤0 or$\;{\mathrm{APCE}}_{t}\geq \;\epsilon_{\mathrm{th}}$, it indicates that the target is coming out of the shelter, and both the tracking and model updates are based on STC.
2. When δ≤0 and$\;{\mathrm{APCE}}_{t}<\epsilon_{\mathrm{th}}$, it indicates that the target is in the occlusion state and tracking is based on the fractional-gain Kalman filter. The tracking model is also updated based on the Kalman filter prediction.
3. When δ>0 or$\;{\mathrm{APCE}}_{t}<\epsilon_{\mathrm{th}}$, it indicates that the target occludes, and both the tracking and model update are based on STC.
4. When δ>0 or$\;{\mathrm{APCE}}_{t}\geq \;\epsilon_{\mathrm{th}}$, it indicates that the target tracking is good and that both the tracking and model update are based on STC.

**Figure 4:**
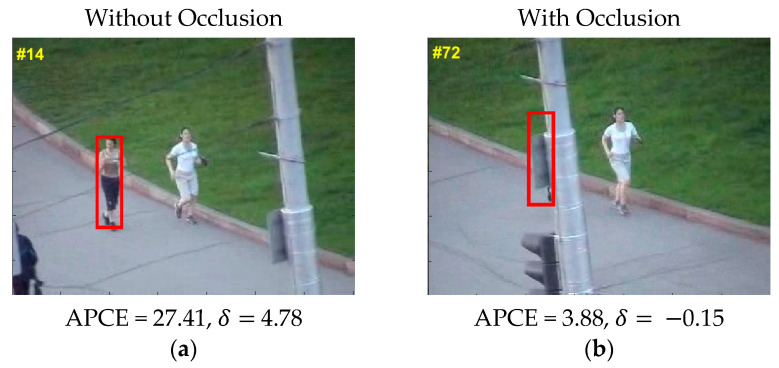
Occlusion detection mechanism. Pictures in the figure are part of OTB-100 dataset [26].

As seen in Figure 4a, without occlusion, both APCE and δ are high; therefore, no occlusion occurs. However, when both APCE and δ give low values, as shown in Figure 4b, case occlusion occurs and the occlusion handling mechanism is activated. By using this mechanism, proposed tracking achieved significant results for the occlusion challenge.

### 3.3. Fractional-Gain Kalman Filter

The Kalman filter is widely used in the research area of VOT. A modified discrete time linear system can be characterized by Equations (19) and (20):

(19) $$x_{k}={\mathrm{Ax}}_{k-1}+{\mathrm{Bu}}_{k}+{\;w}_{k}$$

(20) $$z_{k}={\mathrm{Hx}}_{k}+{\;v}_{k}$$

where $x_{k}$ is the state vector, $z_{k}$ is system output, $u_{k}$ is system input, and $v_{k}$ is output noise. A, B, and H are transition, control, and measurement matrices, respectively. The innovation equation is the difference between the estimated output ${\hat{z}}_{k}\;$and actual output $z_{k}$ defined in (21):

(21) $$\mathrm{innovation}={\;z}_{k}-{\hat{z}}_{k}={\;z}_{k}-{H\hat{x}}_{k}^{-}$$

where ${\hat{x}}_{k}^{-}$ is the priori state. The estimation of the next state ${\hat{x}}_{k}$ with a modified gain is given in (22) and (23):

(22) $${\hat{x}}_{k}={\hat{x}}_{k}^{-}+{\;K}_{\mathrm{new}}\left(\mathrm{innovation}\right)={\hat{x}}_{k}^{-}+{\;K}_{\mathrm{new}}\left(z_{k}-{H\hat{x}}_{k}^{-}\right)\;$$

(23) $$K_{\mathrm{new}}={\;K}_{k}+{\;f}_{k}={\;K}_{k}+\Delta^{\gamma }{\;K}_{k}$$

where $\Delta^{\gamma }{\;K}_{k}$ is the fractional derivative of previous Kalman gain. Priori error e^k− between actual and estimated state and its covariance $P_{k}^{-}$ can be given in (24) and (25):

(24) $${\hat{e}}_{k}^{-}={\;x}_{k}-{\hat{x}}_{k}^{-}$$

(25) $$P_{k}^{-}=E\;\left\{{\left({\hat{e}}_{k}^{-}\right)}^{2}\right\}$$

Posteriori error $e_{k}$ between actual and estimated state and its covariance $P_{k}$ can be given, as in (26) and (27):

(26) $$e_{k}={\;x}_{k}-{\hat{x}}_{k}$$

(27) $$P_{k}=E\;\left\{{\left(e_{k}\right)}^{2}\right\}$$

Kalman gain K is calculated by minimizing posteriori error covariance $P_{k}$ as given in (28):

(28) $$P_{k}=\;E{\left(x_{k}-{\hat{x}}_{k}\right)}^{2}=E{\left({\hat{x}}_{k}-{\hat{x}}_{k}^{-}-\left(K+\Delta^{\gamma }\;K\right)\left(z_{k}-{H\hat{x}}_{k}^{-}\right)\right)}^{2}$$

Finding the value of K in (29):

(29) $$\frac{dE\;{\left({\hat{x}}_{k}-{\hat{x}}_{k}^{-}-\left(K+\Delta^{\gamma }\;K\right)\left(z_{k}-{H\hat{x}}_{k}^{-}\right)\right)}^{2}}{dK}=0$$

$K_{\mathrm{new}}$ can be written as in (30):

(30) $$K_{\mathrm{new}}=K+E\;\left\{\overset{k}{\underset{q=0}{\sum }}{\left(-1\right)}^{q+1}\left(\begin{array}{c} n\\ q \end{array}\right){\;K}_{k-q}\;\right\}$$

The modified Kalman gain $K_{\mathrm{new}}$ consists of two terms. The first term represents the Kalman filter’s gain, and the second represents the mean of the fractional difference of previous gains. The ${\left(-1\right)}^{q+1}$ makes the mean value nominal.

### 3.4. Adaptive Learning Rate

The motion of the target is changed in each frame during tracking. It is, therefore, necessary to update the target model adaptively rather than on a fixed learning rate. We used an APCE-based degree indicator to better cope with environmental changes occurring during tracking to make it adaptive. In the present work, we used maxima of historical APCE values to normalize APCE, since the APCE value is very high. The degree indicator $d_{\mathrm{APCE}}$ is defined in (31):

(31) $$d_{\mathrm{APCE}}=\frac{{\mathrm{APCE}}_{t}}{\max\left(\left\{{\mathrm{APCE}}_{t_{s}},\ldots ,{\mathrm{APCE}}_{t-1}\right\}\right)}$$

where $t_{s}$ is the start index frame. The value of the learning rate is adjusted as in (32):

(32) $$\rho =\{\begin{array}{c} 0.075\;\;\;,{\;d}_{\mathrm{APCE}}>\tau_{\mathrm{th}}\;\;\;\\ 0.075*d_{\mathrm{APCE}}\;,{\;d}_{\mathrm{APCE}}\leq \tau_{\mathrm{th}}\; \end{array}$$

where $\tau_{\mathrm{th}}$ is the threshold value acquired by performing multiple experiments.

**Figure 5:**
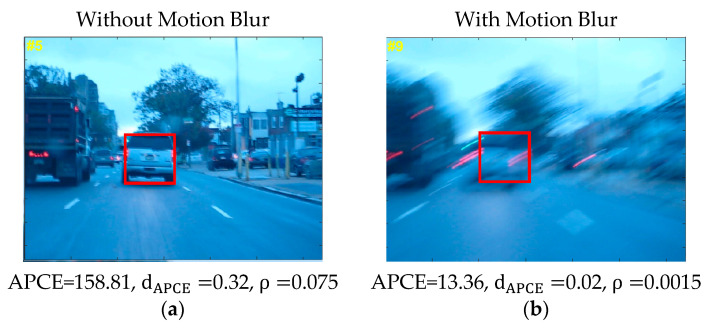
Learning rate mechanism. Pictures in the figure are part of OTB-100 dataset [26].

Figure 5a shows that, without both motion and blur, APCE and $d_{\mathrm{APCE}}$ are high; therefore, the learning rate of tracking should be fast. However, when motion blur occurs, both APCE and $d_{\mathrm{APCE}}$ give low values, as shown in Figure 5b. Thus, in that case, the model should be updated slowly due to the appearance change of the target. By using this mechanism, the proposed tracking achieved significant results for the motion blur challenge. The tracker is given in Algorithm 1.
**Algorithm 1:** Proposed Tracking Method  **Input**: Video with initialized ground truth on frame 1.  **Output:** Rectangle on each frame. ***for*** 1st to the last frame.  Compute context prior model by using (3).  Compute confidence map by using (11).  Compute center of target location.  Estimate scale by using (15).  Compute APCE by using (16).  Determine occlusion detection using (17) and (18).  Check four rules of occlusion detection given in Section 3.2.  **if** rule 2 occurs     Activate fractional-gain Kalman filter  Compute fractional Kalman gain by using (30).  Predict position by using (22).  Compute error covariance by using (28).  **end**  Calculate occlusion indicator using (31).  Calculate learning rate using (32)  Update context prior model by using (3).  Update spatial context model by using (9).  Update STC model by using (12).  Estimate the position of target. ***End***

## 4. Performance Analysis

Comprehensive assessments were conducted on videos taken from the OTB 2015 [26] dataset for the proposed tracking method’s quantitative and quantitative evaluation. These sequences include scale variations, motion blur, and fast motion challenges.

### 4.1. Evaluation Criteria

The proposed algorithm is compared with tracking methods on two evaluation criteria: distance precision rate (DPR) and center location error (CLE). The calculation formula for CLE is mentioned in (33):

(33) $$\mathrm{CLE}\;=\sqrt{{\left(x_{i}-x_{\mathrm{gt}}\right)}^{2}+{\left(y_{i}-y_{\mathrm{gt}}\right)}^{2}}$$

### 4.2. Quantitative Analysis

DPR evaluation is presented in Table 1. In videos Blurcar1, Car2, Human7, Jogging1, and Jogging2, the proposed algorithm outperforms Modified KCF, MOSSE_CA_, MACF, KCF_MTSA, and STC. For the sequences Blurcar3, Blurcar4, Boy, Dancer2, and Suv, the proposed tracker has marginally less precision value. Overall, the proposed algorithm has a higher mean value than the other algorithms.

Average center location error evaluation is presented in Table 2. In the videos Blurcar1, Car2, Dancer2, Jogging1, and Human7, the proposed algorithm outperforms Modified KCF, MOSSE_CA_, MACF, KCF_MTSA, and STC. For the videos Blurcar3, Blurcar4, Boy, Jogging2, and Suv, the proposed algorithm has marginally high error values. Overall, the proposed algorithm has the lowest mean error compared to the other algorithms.

**Figure 6:**
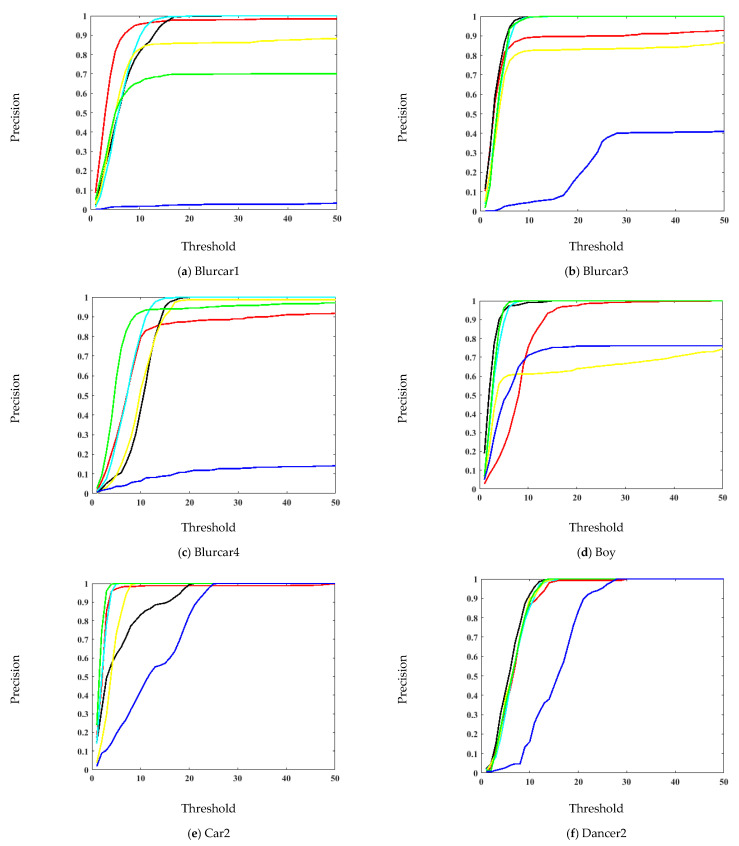

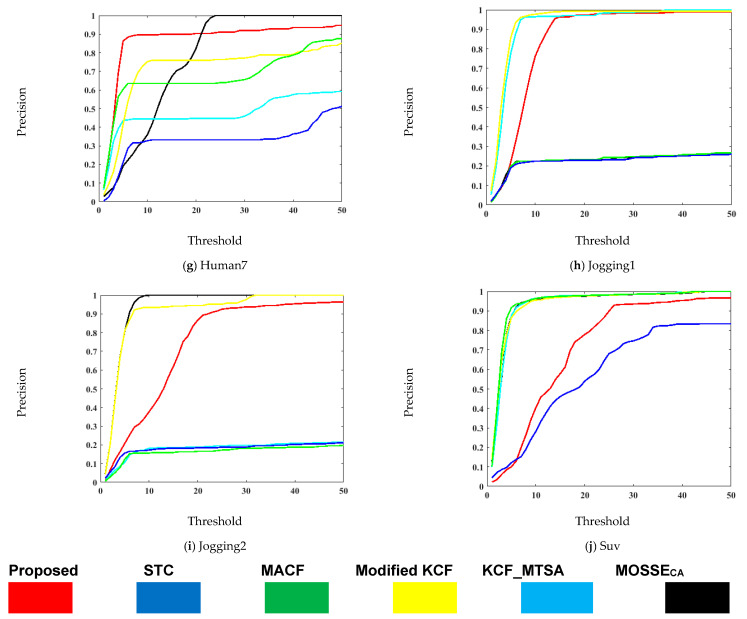
Precision plot comparison for the OTB-100 dataset [26].

**Figure 7:**
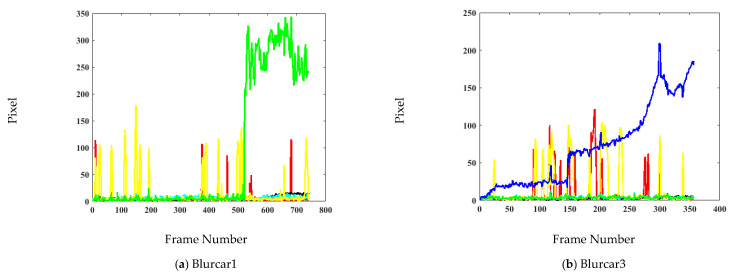

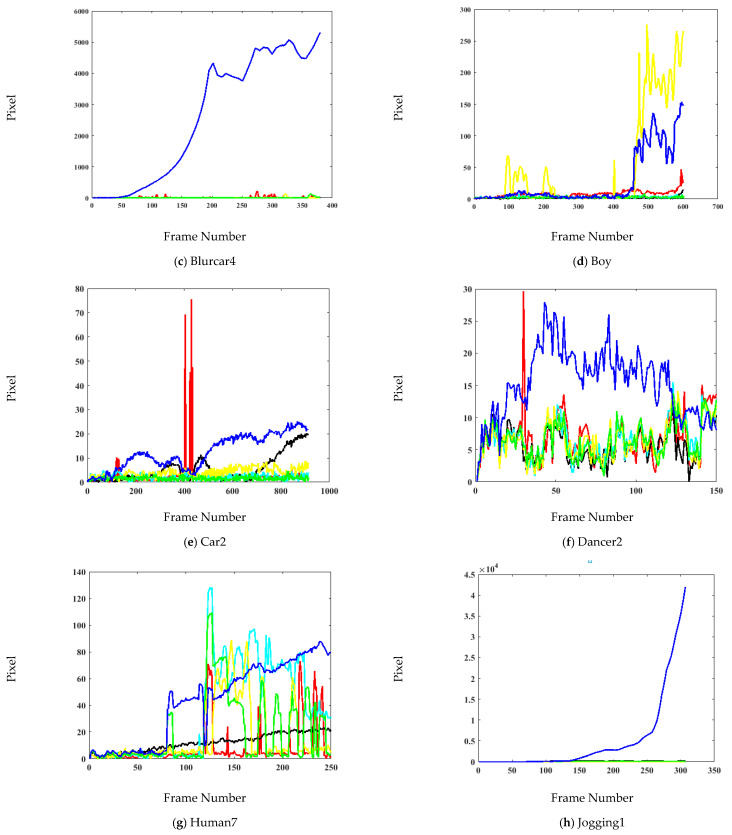

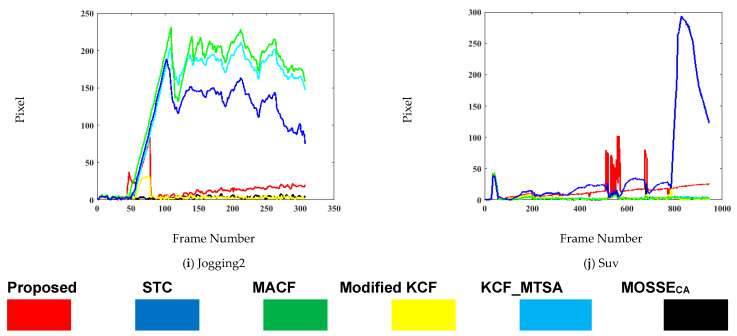
Center location error (in pixels) comparison for the OTB-100 dataset [26].

The precision and error plots are presented in Figure 6 and Figure 7, respectively. These plots provide a frame-by-frame comparison in entire image sequences. Since precision and location error gives the mean of the entire sequence, it is possible that the algorithm loses the target for a few frames but correctly tracks again. Therefore, these plots were presented to show the effectiveness of the tracking method. In the videos Blurcar1, Human7, Jogging1, and Jogging2, the proposed algorithm has the highest precision in the entire video. It has slightly low accuracy in the Blurcar3, Blurcar4, Boy, Car2, Dancer2 and Suv videos. The proposed algorithm has the lowest error in the Blurcar1, Human7, Jogging1, and Jogging2 videos. It has marginally high error compared with a few trackers for the Blurcar3, Blurcar4, Boy, Car2, Dancer2, and Suv sequences.

Frames per second (fps) analysis is presented in Table 3. In the Blurcar1, Car2, Dancer2, Human7, and Jogging1 videos, the proposed algorithm outperforms Modified KCF, MOSSE_CA_, MACF, KCF_MTSA, and STC in terms of precision in error at the expense of modest frame rate.

The computational time for the learning rate module is presented in Table 4. It can be seen that the proposed tracker takes less time in motion blur sequences. However, the overall speed of the tracker is slightly slow, given in Table 3. Combining the different tracking modules presented in Section 3, performance of the proposed tracker is significant as each module is specifically designed and incorporated into the STC framework, making it efficient in terms of less error and high precision for different challenging attributes in VOT.

### 4.3. Qualitative Analysis

**Figure 8:**
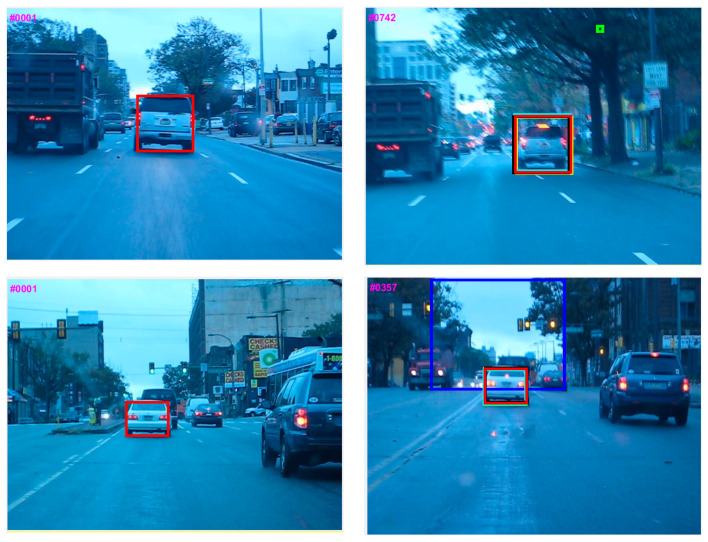

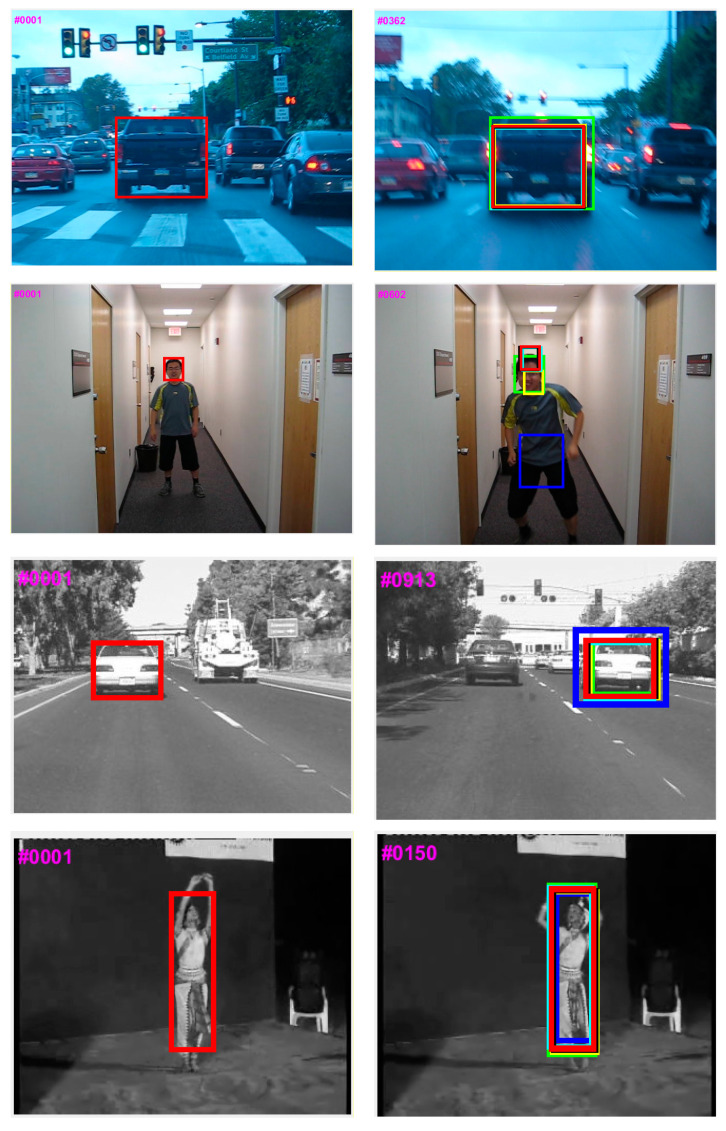

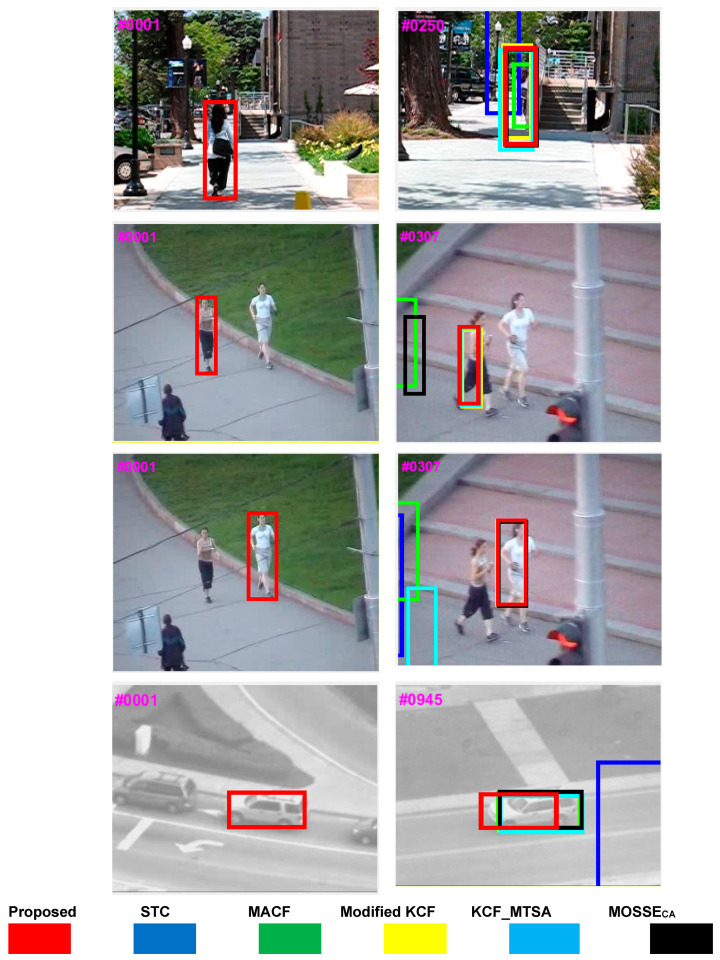
Qualitative comparison for the OTB-100 dataset [26].

Figure 8 depicts the qualitative analysis of the proposed tracking with five state-of-the-art trackers. Modified KCF and KCF_MTSA are extensions of KCF [62] based tracking methods. However, Modified KCF is not robust to motion blur (Blurcar1, Blurcar3, and Human7), whereas the performance of KCF_MTSA is affected in occlusion (Jogging2) and motion blur (Human7). MACF is an improved version of fast discriminative scale space tracking [63] and achieved favorable results in various challenges of VOT. However, it does not perform well in motion blur (Blurcar1) and occlusion (Jogging1 and Jogging 2). MOSSE_CA_ is an improved context-aware formulation version of the MOSSE [64] tracker. The results are exceptional except in the Jogging1 and Human7 sequences. STC is the baseline tracker of the proposed method and achieves favorable results. However, it can be seen that it does not address occlusion (Jogging1 and Jogging2) or motion blur (Blurcar1, Blurcar3, Blurcar4, Boy, and Human7).

It can be seen that the proposed tracker outperforms other tracking methods in these sequences. This performance is attributed to three factors. First, a max-pooling-based scale scheme is incorporated, making it less sensitive to scale variations (Boy). Second, incorporation of the APCE-based modified occlusion detection mechanism and fractional-gain Kalman filter-based occlusion handling makes it effective toward occlusions (Jogging1, Jogging2, and Suv). Third, the combination of APCE criteria in the learning rate of the proposed algorithm model update effectively, making it efficient towards motion blur (Blurcar1, Blurcar3, Blurcar4, Boy, and Human7) and illumination variations (Car2 and Dancer2).

## 5. Discussion

We discuss several observations from performance analysis. First, max-pooling-based scale formulation in spatiotemporal context outperforms trackers without this formulation. This can be attributed to estimating maximum likelihood by using target appearance sampled at a different set of scales. Second, trackers which utilize modules for occlusion detection and handling module outperform trackers without these modules. This can be attributed to the fractional-gain Kalman filter and an APCE-based occlusion detection mechanism preventing tracker from drift. Third, trackers with adaptive learning rate perform better than those with fixed learning rate.

## 6. Conclusions

This paper contributes insight into an STC-based accurate tracking algorithm by incorporating max-pooling, fractional-gain Kalman, and APCE measures for occlusion detection and tracking model update. It can improve the adaptability of the target model and prevent error accumulation. Evaluations specify that the proposed tracker achieves enhanced results in various complicated scenarios. However, there are some problems: (1) tracking performance is severely affected in dense occlusion; (2) the tracker lost the target of interest in deformation and fast motion; and (3) frame rate of the proposed tracking method is slow. These three points will be the focus of follow-up research. Additionally, considering the challenges of VOT, we also plan to perform future in-depth research on the fusion of features and better prediction estimation mechanisms, and carry out Raspberry Pi, FPGA, and DSP-based hardware implementation and practical application for meeting the requirements of society.

## Figures and Tables

**Table 1:** Distance precision rate.

Sequence	Proposed	Modified KCF [59]	STC [27]	MACF [57]	MOSSE_CA_ [58]	KCF_MTSA [41]
Blurcar1	0.978	0.858	0.024	0.698	0.999	0.999
Blurcar3	0.896	0.829	0.406	1	1	1
Blurcar4	0.876	0.987	0.113	0.944	1	1
Boy	0.973	0.64	0.761	1	1	1
Car2	0.988	1	1	1	0.993	1
Dancer2	0.993	1	1	1	1	1
Human7	0.904	0.76	0.332	0.636	0.824	0.448
Jogging1	0.973	0.993	0.228	0.231	0.231	0.964
Jogging2	0.866	0.945	0.186	0.166	1	0.189
Suv	0.778	0.978	0.805	0.978	0.976	0.98
**Mean Precision**	**0.923**	**0.899**	**0.486**	**0.765**	**0.902**	**0.858**

**Table 2:** Average center location error.

Sequence	Proposed	Modified KCF [59]	STC [27]	MACF [57]	MOSSE_CA_ [58]	KCF_MTSA [41]
Blurcar1	4.86	16.05	1.31 × 10^6^	85.16	6.34	6.01
Blurcar3	9.12	14.46	71.37	3.69	2.98	3.7
Blurcar4	15.01	11.19	2.61 × 10^3^	8.04	10.15	7.15
Boy	8.09	50.34	27.4	2.65	2.31	2.91
Car2	2.68	3.96	12.43	1.55	5.39	2.13
Dancer2	6.82	6.41	15.3	6.48	5.8	6.68
Human7	7.59	16.74	42.98	19.62	12.14	36.63
Jogging1	8.39	3.72	5010	94.97	115.98	4.27
Jogging2	14.2	4.74	104.02	147.77	3.47	136.4
Suv	15.36	3.65	48	3.34	3.73	3.71
**Mean Error**	**9.212**	**13.126**	**1.3 × 10^6^**	**37.327**	**16.829**	**20.959**

**Table 3:** Frames per second (fps).

Sequence	Proposed	Modified KCF [59]	STC [27]	MACF [57]	MOSSE_CA_ [58]	KCF_MTSA [41]
Blurcar1	10.78	66.29	27.75	18.5	53.06	15.35
Blurcar3	18.04	33.62	28.87	32.7	51.74	6.08
Blurcar4	5.7	21.42	20.07	8.64	27.65	5.83
Boy	26.67	85.51	33.48	58.7	157.17	22.02
Car2	57.18	90.7	94.08	55.3	95.38	11.2
Dancer2	29.66	29.65	65.1	29.2	38.87	6.26
Human7	25.17	34.44	59.66	40.5	26.11	11.48
Jogging1	42.71	95.45	61.75	49	36.59	12.55
Jogging2	22.77	33.01	56.92	34.6	33.97	11
Suv	69.61	76.32	98.03	50.9	79.7	8.44

**Table 4:** Computation time of the proposed tracker’s learning rate module.

Sequence	Frame Size	Number of Frames	Time
Blurcar1	640 × 480	742	0.011
Blurcar3	640 × 480	357	0.008
Blurcar4	640 × 480	380	0.009
Boy	640 × 480	602	0.009
Car2	320 × 240	913	0.018
Dancer2	320 × 262	150	0.006
Human7	320 × 240	250	0.007
Jogging1	352 × 288	307	0.012
Jogging2	352 × 288	307	0.008
Suv	320 × 240	945	0.017

## Data Availability

Data are contained within the article.

## References

[1] Pantrigo J.J., Hernández J., Sánchez A. (2010). Multiple and variable target visual tracking for video-surveillance applications. Pattern Recognit. Lett..

[2] Ahmed I., Jeon G. (2021). A real-time person tracking system based on SiamMask network for intelligent video surveillance. J. Real-Time Image Process.

[3] Carcagnì P., Mazzeo P.L., Distante C., Spagnolo P., Adamo F., Indiveri G. (2014). A UAV-Based Visual Tracking Algorithm for Sensible Areas Surveillance. Proceedings of the International Workshop on Modelling and Simulation for Autonomous Systems.

[4] Geiger A., Lauer M., Wojek C., Stiller C., Urtasun R. (2013). 3d traffic scene understanding from movable platforms. IEEE Trans. Pattern Anal. Mach. Intell..

[5] Wang N., Shi J., Yeung D.-Y., Jia J. (2015). Understanding and diagnosing visual tracking systems. Proceedings of the IEEE International Conference on Computer Vision.

[6] Bonatti R., Ho C., Wang W., Choudhury S., Scherer S. (2019). Towards a robust aerial cinematography platform: Localizing and tracking moving targets in unstructured environments. Proceedings of the 2019 IEEE/RSJ International Conference on Intelligent Robots and Systems (IROS).

[7] Petitti A., di Paola D., Milella A., Mazzeo P.L., Spagnolo P., Cicirelli G., Attolico G. (2013). A distributed heterogeneous sensor network for tracking and monitoring. Proceedings of the 2013 10th IEEE International Conference on Advanced Video and Signal Based Surveillance.

[8] Wu Y., Lim J., Yang M.-H. (2013). Online object tracking: A benchmark. Proceedings of the IEEE Conference on Computer Vision and Pattern Recognition.

[9] Danelljan M., Khan F.S., Felsberg M., Granström K., Heintz F., Rudol P., Wzorek M., Kvarnström J., Doherty P. (2014). A low-level active vision framework for collaborative unmanned aircraft systems. Proceedings of the European Conference on Computer Vision.

[10] Petitti A., di Paola D., Milella A., Mazzeo P.L., Spagnolo P., Cicirelli G., Attolico G. (2014). A heterogeneous robotic network for distributed ambient assisted living. Human Behavior Understanding in Networked Sensing.

[11] Amorim T.G.S., Souto L.A., Nascimento T.P.D., Saska M. (2021). Multi-Robot Sensor Fusion Target Tracking with Observation Constraints. IEEE Access.

[12] Ali A., Kausar H., Muhammad I.K. (2009). Automatic visual tracking and firing system for anti aircraft machine gun. Proceedings of the 6th International Bhurban Conference on Applied Sciences & Technology.

[13] Cao J., Song C., Song S., Xiao F., Zhang X., Liu Z., Ang M.H. (2021). Robust Object Tracking Algorithm for Autonomous Vehicles in Complex Scenes. Remote Sens..

[14] Lee Y., Lee S., Yoo J., Kwon S. (2021). Efficient Single-Shot Multi-Object Tracking for Vehicles in Traffic Scenarios. Sensors.

[15] Ali A., Jalil A., Niu J., Zhao X., Rathore S., Ahmed J., Iftikhar M.A. (2016). Visual object tracking—Classical and contemporary approaches. Front. Comput. Sci..

[16] Mazzeo P.L., Spagnolo P., Distante C. (2015). Visual Tracking by using dense local descriptors. Adaptive Optics: Analysis, Methods & Systems.

[17] Ali A., Jalil A., Ahmed J. (2016). A new template updating method for correlation tracking. Proceedings of the 2016 International Conference on Image and Vision Computing New Zealand (IVCNZ).

[18] Abbasi S., Rezaeian M. (2021). Visual object tracking using similarity transformation and adaptive optical flow. Multimed. Tools Appl..

[19] Adamo F., Mazzeo P.L., Spagnolo P., Distante C. (2015). A FragTrack algorithm enhancement for total occlusion management in visual object tracking. Automated Visual Inspection and Machine Vision.

[20] Yang L., Zhong-li W., Bai-gen C. (2014). An intelligent vehicle tracking technology based on SURF feature and Mean-shift algorithm. Proceedings of the 2014 IEEE International Conference on Robotics and Biomimetics (ROBIO 2014).

[21] Matsushita Y., Yamaguchi T., Harada H. (2019). Object tracking using virtual particles driven by optical flow and Kalman filter. Proceedings of the 2019 19th International Conference on Control, Automation and Systems (ICCAS).

[22] Mei X., Ling H. (2011). Robust visual tracking and vehicle classification via sparse representation. IEEE Trans. Pattern Anal. Mach. Intell..

[23] Judy M., Poore N.C., Liu P., Yang T., Britton C., Bolme D.S., Mikkilineni A.K., Holleman J. (2018). A digitally interfaced analog correlation filter system for object tracking applications. IEEE Trans. Circuits Syst. I Regul. Pap..

[24] Adamo F., Carcagnì P., Mazzeo P.L., Distante C., Spagnolo P. (2014). TLD and Struck: A Feature Descriptors Comparative Study. International Workshop on Activity Monitoring by Multiple Distributed Sensing.

[25] Dong E., Deng M., Tong J., Jia C., Du S. (2019). Moving vehicle tracking based on improved tracking–learning–detection algorithm. IET Comput. Vis..

[26] Wu Y., Lim J., Yang M. (2015). Object Tracking Benchmark. IEEE Trans. Pattern Anal. Mach. Intell..

[27] Zhang K., Zhang L., Liu Q., Zhang D., Yang M.H. (2014). Fast visual tracking via dense spatio-temporal context learning. Lecture Notes in Computer Science (Including Subseries Lecture Notes in Artificial Intelligence and Lecture Notes in Bioinformatics).

[28] Die J., Li N., Liu Y., Wu Y. (2019). Correlation Filter Tracking Algorithm Based on Spatio-Temporal Context. Proceedings of the International Conference on Natural Computation, Fuzzy Systems and Knowledge Discovery.

[29] Yang X., Zhu S., Zhou D., Zhang Y. (2020). An improved target tracking algorithm based on spatio-temporal context under occlusions. Multidimens. Syst. Signal Process..

[30] Zhang Y., Wang L., Qin J. (2019). Adaptive spatio-temporal context learning for visual tracking. Imaging Sci. J..

[31] Zhang D., Dong E., Yu H., Jia C. (2020). An Improved Object Tracking Algorithm Combining Spatio-Temporal Context and Selection Update. Proceedings of the 2020 Chinese Automation Congress (CAC).

[32] Song H., Wu Y., Zhou G. (2021). Design of bio-inspired binocular UAV detection system based on improved STC algorithm of scale transformation and occlusion detection. Int. J. Micro Air Veh..

[33] Feng F., Shen B., Liu H. (2018). Visual object tracking: In the simultaneous presence of scale variation and occlusion. Syst. Sci. Control Eng..

[34] Li J., Zhou X., Chan S., Chen S. (2017). Robust object tracking via large margin and scale-adaptive correlation filter. IEEE Access.

[35] Zhang M., Xing J., Gao J., Hu W. (2015). Robust visual tracking using joint scale-spatial correlation filters. Proceedings of the 2015 IEEE International Conference on Image Processing (ICIP).

[36] Nguyen A.H., Mai L., Do H.N. (2020). Visual Object Tracking Method of Spatio-temporal Context Learning with Scale Variation. Proceedings of the International Conference on the Development of Biomedical Engineering in Vietnam.

[37] Wang X., Hou Z., Yu W., Pu L., Jin Z., Qin X. (2018). Robust occlusion-aware part-based visual tracking with object scale adaptation. Pattern Recognit..

[38] Ma H., Lin Z., Acton S.T. (2019). FAST: Fast and Accurate Scale Estimation for Tracking. IEEE Signal Process. Lett..

[39] Danelljan M., Häger G., Khan F., Felsberg M. (2014). Accurate scale estimation for robust visual tracking. Proceedings of the British Machine Vision Conference.

[40] Li Y., Zhu J. (2014). A scale adaptive kernel correlation filter tracker with feature integration. Proceedings of the European conference on computer vision.

[41] Bibi A., Ghanem B. (2015). Multi-template scale-adaptive kernelized correlation filters. Proceedings of the IEEE International Conference on Computer Vision Workshops.

[42] Lu H., Xiong D., Xiao J., Zheng Z. (2020). Robust long-term object tracking with adaptive scale and rotation estimation. Int. J. Adv. Robot. Syst..

[43] Yin X., Liu G., Ma X. (2020). Fast Scale Estimation Method in Object Tracking. IEEE Access.

[44] Ma H., Acton S.T., Lin Z. (2020). SITUP: Scale invariant tracking using average peak-to-correlation energy. IEEE Trans. Image Process..

[45] Mehmood K., Jalil A., Ali A., Khan B., Murad M., Khan W.U., He Y. (2021). Context-Aware and Occlusion Handling Mechanism for Online Visual Object Tracking. Electronics.

[46] Khan B., Ali A., Jalil A., Mehmood K., Murad M., Awan H. (2020). AFAM-PEC: Adaptive Failure Avoidance Tracking Mechanism Using Prediction-Estimation Collaboration. IEEE Access.

[47] Mehmood K., Jalil A., Ali A., Khan B., Murad M., Cheema K.M., Milyani A.H. (2021). Spatio-Temporal Context, Correlation Filter and Measurement Estimation Collaboration Based Visual Object Tracking. Sensors.

[48] Yang H., Wang J., Miao Y., Yang Y., Zhao Z., Wang Z., Sun Q., Wu D.O. (2019). Combining Spatio-Temporal Context and Kalman Filtering for Visual Tracking. Mathematics.

[49] Ali A., Mirza S.M. (2006). Object tracking using correlation, Kalman filter and fast means shift algorithms. Proceedings of the 2006 International Conference on Emerging Technologies.

[50] Kaur H., Sahambi J.S. (2015). Vehicle tracking using fractional order Kalman filter for non-linear system. Proceedings of the International Conference on Computing, Communication & Automation.

[51] Soleh M., Jati G., Hilman M.H. (2018). Multi Object Detection and Tracking Using Optical Flow Density–Hungarian Kalman Filter (Ofd-Hkf) Algorithm for Vehicle Counting. J. Ilmu Komput. dan Inf..

[52] Farahi F., Yazdi H.S. (2020). Probabilistic Kalman filter for moving object tracking. Signal Process. Image Commun..

[53] Gunjal P.R., Gunjal B.R., Shinde H.A., Vanam S.M., Aher S.S. (2018). Moving object tracking using kalman filter. Proceedings of the 2018 International Conference on Advances in Communication and Computing Technology (ICACCT).

[54] Ali A., Jalil A., Ahmed J., Iftikhar M.A., Hussain M. (2015). Correlation, Kalman filter and adaptive fast mean shift based heuristic approach for robust visual tracking. Signal Image Video Process..

[55] Kaur H., Sahambi J.S. (2016). Vehicle tracking in video using fractional feedback Kalman filter. IEEE Trans. Comput. Imaging.

[56] Zhou X., Fu D., Shi Y., Wu C. (2017). Adaptive Learning Compressive Tracking Based on Kalman Filter. Proceedings of the International Conference on Image and Graphics.

[57] Zhang Y., Yang Y., Zhou W., Shi L., Li D. (2018). Motion-aware correlation filters for online visual tracking. Sensors.

[58] Mueller M., Smith N., Ghanem B. (2017). Context-aware correlation filter tracking. Proceedings of the IEEE Conference on Computer Vision and Pattern Recognition.

[59] Shin J., Kim H., Kim D., Paik J. (2020). Fast and robust object tracking using tracking failure detection in kernelized correlation filter. Appl. Sci..

[60] Sierociuk D., Dzieliński A. (2006). Fractional Kalman filter algorithm for the states, parameters and order of fractional system estimation. Int. J. Appl. Math. Comput. Sci..

[61] Wang M., Liu Y., Huang Z. (2017). Large margin object tracking with circulant feature maps. Proceedings of the IEEE Conference on Computer Vision and Pattern Recognition.

[62] Henriques J.F., Caseiro R., Martins P., Batista J. (2014). High-speed tracking with kernelized correlation filters. IEEE Trans. Pattern Anal. Mach. Intell..

[63] Danelljan M., Häger G., Khan F.S., Felsberg M. (2016). Discriminative scale space tracking. IEEE Trans. Pattern Anal. Mach. Intell..

[64] Bolme D.S., Beveridge J.R., Draper B.A., Lui Y.M. (2010). Visual object tracking using adaptive correlation filters. Proceedings of the 2010 IEEE Computer Society Conference on Computer Vision and Pattern Recognition.

